# Renewable energy production and demand dataset for the energy system of Colombia

**DOI:** 10.1016/j.dib.2019.105084

**Published:** 2020-01-03

**Authors:** Oscar Pupo-Roncallo, Javier Campillo, Derek Ingham, Kevin Hughes, Mohammed Pourkashanian

**Affiliations:** a Energy 2050, The University of Sheffield, Ella Armitage Building, Sheffield, S37RD, UK; b Electrical Engineering Department, Universidad Tecnológica de Bolívar, Parque Industrial y Tecnológico Carlos Vélez Pombo, Cartagena, 130 012, Colombia

**Keywords:** Wind power, Solar power, Electricity demand, Renewable energy, Colombia

## Abstract

During the last decades, an increasing number of studies have focused their attention on the development of energy system models in order to facilitate sustainable energy planning strategies and understand the technical challenges associated with the integration of renewable energy sources. However, these models usually require detailed and large amount of data as inputs. The data presented in this article provides key inputs and modelling assumptions adopted in the research paper titled “Large scale integration of renewable energy sources (RES) in the future Colombian energy system” [1]. These datasets can be used by researchers and policymakers in order to analyse different pathways oriented to the development of low carbon strategies for Colombia and countries with similar energy systems.

**Table:** Specifications Table

Subject	Renewable Energy, Sustainability and the Environment
Specific subject area	Renewable energy assessment
Type of data	Tables, graphs and maps
How data were acquired	Databases of international and domestic organisations.
Data format	Raw, filtered and analysed
Parameters for data collection	Data selected based on inputs required to build a high resolution energy system model and the sites with high potential for renewable energy generation in Colombia [1].
Description of data collection	Data was gathered from online databases of international organisations, the Colombian national grid and government agencies.
Data source location	Country: Colombia
Data accessibility	Repository: Mendeley Data Data identification number: w6cwkn4jm7.2 URL to data: https://doi.org/10.17632/w6cwkn4jm7.2
Related research article	O. Pupo-Roncallo, J. Campillo, D. Ingham, K. Hughes, M. Pourkashanian. Large scale integration of renewable energy sources (RES) in the future Colombian energy system. Energy, Vol 186, 2019 [1]. DOI of original article: https://doi.org/10.1016/j.energy.2019.07.135

**Table:** 

**Value of the Data** • These datasets can be used for developing a high temporal resolution energy model for Colombia and assess the effects of renewable energy sources integration in future scenarios. In addition, researchers could use the data to replicate the results reported in Ref. [1] and compare them with their model outputs. • The scientific community and policymakers can use the data for the development of low carbon strategies for the Colombian energy system and countries with similar characteristics. • Analysis of the dataset could assist in the understanding of the technical challenges of renewable energy integration into electricity systems. • The complementarity of renewable energy sources for power generation can be evaluated by analysing the dataset. Additionally, the variability of the renewable energy generation during periods of weather abnormalities can be assessed using the distributions provided in this article.

## 1 Data description

The data provided in this paper was used for the development and analysis of a high temporal resolution model for the energy system of Colombia [1]. The dataset presented were collected from domestic organisations, the national grid and international agencies. It includes hourly electricity demand from 2012 to 2016, hourly renewable energy sources (RES) electricity production, time series of natural water inflows and energy exports and imports.

The datasets are organised as explained in Table 1. All the files were made open access in the Mendeley data repository and can be accessed on the following link: https://doi.org/10.17632/w6cwkn4jm7.2 [2].

**Table 1:** Description of items and datasets in the Mendeley data repository [2].

Item	Folder	Description of content
1.	Electricity demand dataset	Hourly electricity demand distributions from 2012 to 2016 are included in this folder. Each file contains 8784 demand data (in MW) for each year and thus considers the additional load hours for leap years. The files are in text (.txt) format.
2.	Water inflows time series	This folder contains time series (2006–2016) of daily natural water inflows. Hourly energy input to the hydropower dams are included in each text file. A figure shows the distribution of these dams in the country and their capacities. Furthermore, hourly average hydro generation distributions are provided for 2014 and the periods affected by El Niño and La Niña southern oscillation (ENSO). A table with the historical installed capacity by fuel type (2006–2016) is also provided.
3.	RES dataset	This folder provides hourly solar and wind power output distributions during normal years and periods affected by ENSO. A map shows the locations where existing and potential plants may be built according to the Colombian electrical information system (SIEL) [3]. The associated coordinates for these plants are also supplied.
4.	Electricity exports-imports dataset	Hourly electricity export and import distributions from 2012 to 2016 are included in this folder. Each file contains 8784 data (in MW) for each year and also considers the additional hours for leap years. In addition, a figure with the electrical network layout in 2016 is included.

### 1.1 Electricity supply and demand

**Fig. 1:**
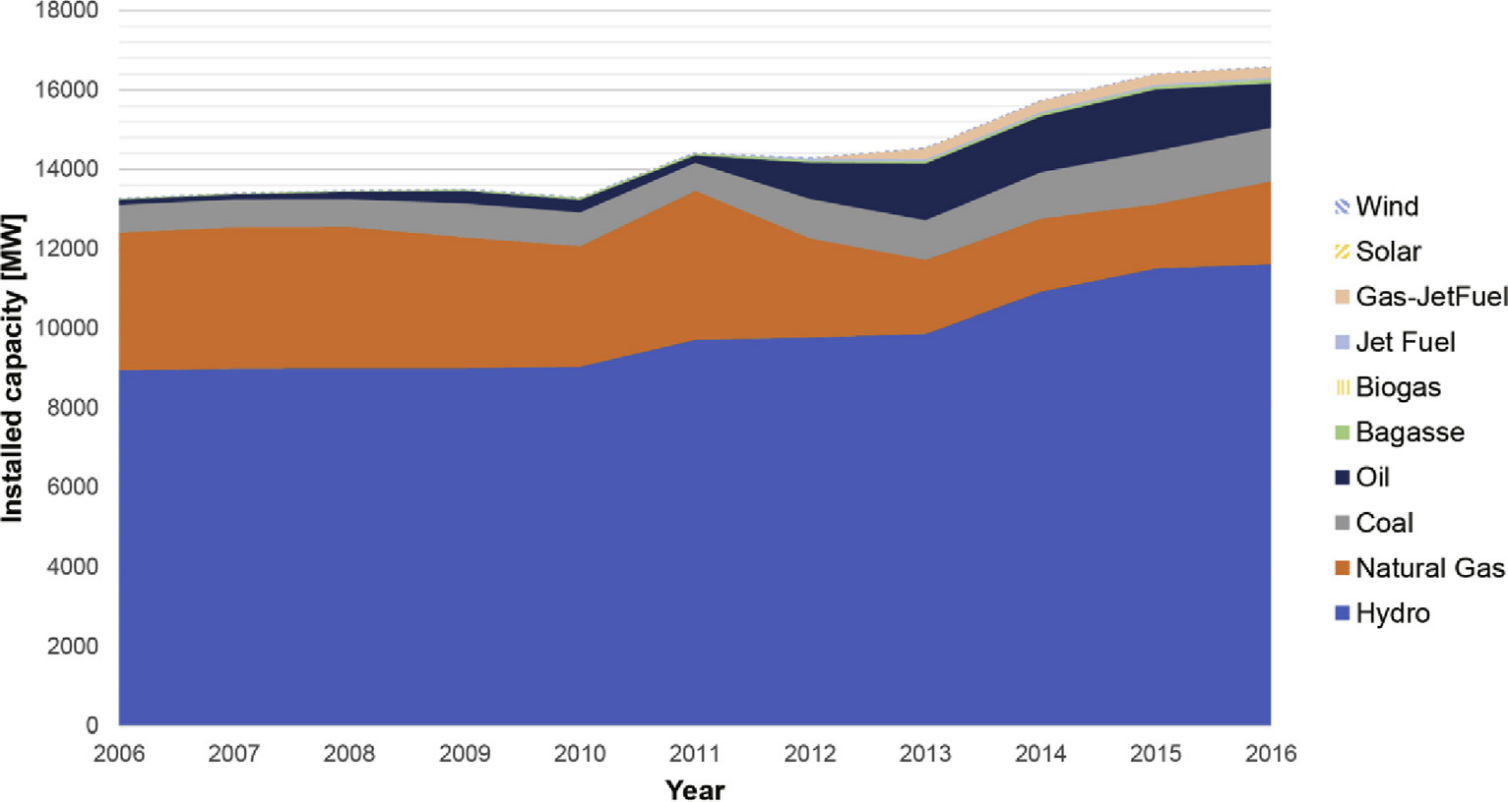
Historical installed capacity by fuel type [5].

**Fig. 2:**
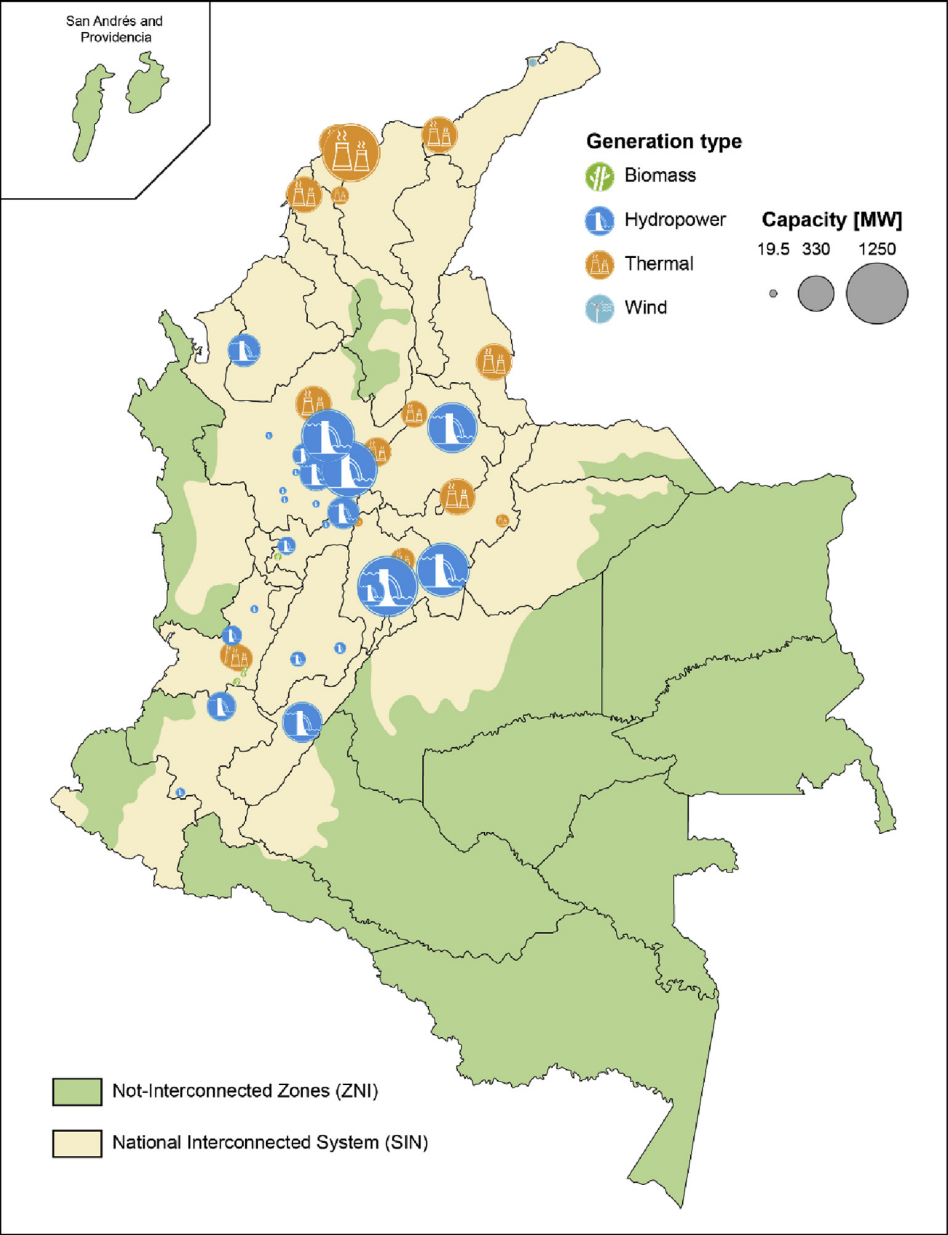
Grid-connected (SIN) areas and power plant locations in 2014. Authors' figure based on [6,7].

The electricity sector in Colombia has been traditionally dominated by hydro and thermal generation, with average energy production of 71% and 28%, respectively. Other renewables sources, such as wind and bioenergy, account for the remaining 1% of the total annual average generation [4]. Fig. 1 shows the installed capacity by fuel type from 2006 to 2016. The geographical distribution of the demand and power units is highly dependent on the availability of the resource. Fig. 2 illustrates the location of conventional and renewable power plants with an installed capacity greater than 19.5 MW in 2014. In addition, the rural regions without access to electricity or Not-Interconnected Zones (ZNI) and regions in the National Interconnected System (SIN) are also shown in Fig. 2.

**Fig. 3:**
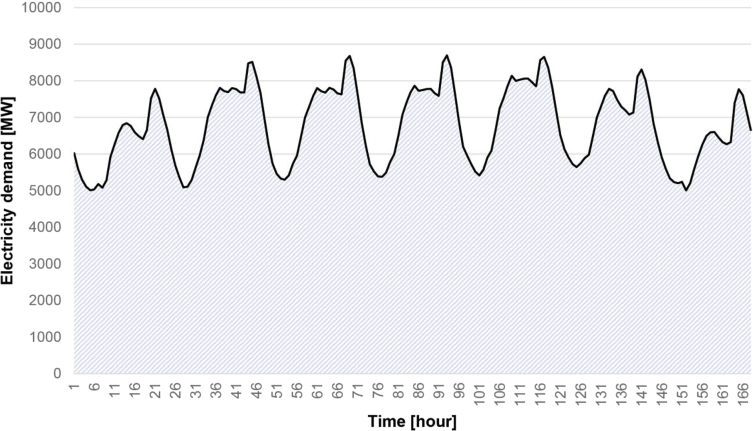
Electricity load in a typical week (Sunday to Saturday) in 2014.

The national electricity demand in a typical week in 2014 can be seen in Fig. 3. The data represents the load behaviour from Sunday to Saturday and the peak and minimum power requirements are evidenced.

## 2 Experimental design, materials, and methods

The energy system model built in Ref. [1] was developed based on Colombian statistics. Hourly electricity demand and production datasets, natural water inflows time series and exports/imports data were collected from XM (National grid company) through its PORTAL BI [8]. Then the data was filtered, organised and classified to generate the distributions described in Table 1. The distributions for years with the presence of weather abnormalities were built using average generation data from the periods 2007–08, 2010–11 in the cases of ENSO “La Niña”; and from the periods 2009–10, 2015–16 in the cases of ENSO “El Niño”.

### 2.1 Renewable energy generation dataset

**Fig. 4:**
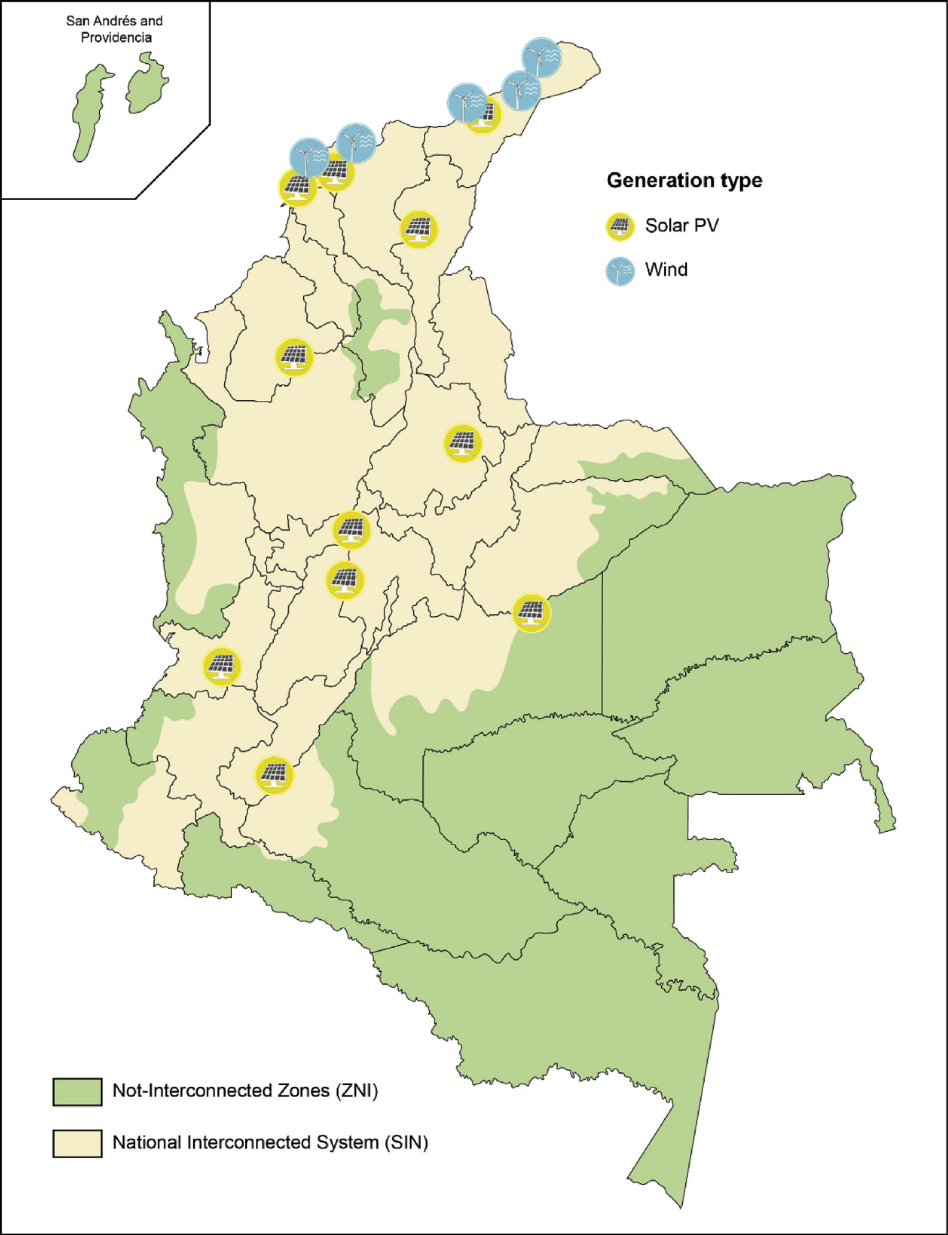
RES generation sites registered in SIEL [3].

The RES datasets for wind and solar energy were built using meteorological data by considering current and future generation sites. Fig. 4 shows the locations considered in Ref. [1] in order to build the hourly distributions. Currently, there is only one wind farm in the country and this has an installed capacity of 19.5 MW [5]. The total installed capacity of solar photovoltaic (PV) in 2019 is estimated to be 17.96 MW with two projects connected to the national grid (Celsia solar Yumbo and Celsia solar Bolivar) [7]. Future generation sites and capacities were taken from the list of projects registered in SIEL [3]. The following sections describe the methodology applied in order to estimate the wind (Section 2.1.1) and solar PV (Section 2.1.2) energy outputs.

#### 2.1.1 Wind power

The wind power output was estimated using long period (over 5 years) average hourly wind speed data. Hourly wind speed at each location was computed and extrapolated to the turbine hub height using the Power Law:

(1) $$v_{H}={\left(\frac{z_{H}}{z_{r}}\right)}^{\alpha }v_{r}$$

where $v_{H}$ and $v_{r}$ are wind velocities at the hub height $z_{H}$ and reference height $z_{r}$, respectively. The power law index (α) is assumed constant and its value for open land with only softly rounded hills is used (α = 0.143) [9]. The reference height is usually 10 m above sea level and meteorological data at this height was supplied by IDEAM and the National Renewable Energy Laboratory (NREL) database [10].

From the characteristics of a standard and commercially available wind turbine (Vestas V90-1.8/2 MW and hub height 95 m [11]), the hourly wind power output at each location (see Fig. 4) was estimated. Then, these results were aggregated and weighted based on their installed capacity in order to obtain the total hourly wind power output. Finally, the model output was validated against the actual generation of the existing wind farm (Jepirachi project) and the percentage difference was approximately 0.32%. Additional details of the model validation process can be found in Ref. [1].

#### 2.1.2 Solar PV power

The solar PV power output requires hourly incident irradiance and module temperature data. The major sites considered for these calculations are shown in Fig. 4. In order to obtain the hourly power generation at each location, the authors have used the model described by Hund et al. in Ref. [12]. The crystalline silicon cells (c-Si) modules are assumed as the technology used and the model is given by the following equation:

(2) $$P\left(G^{*},T^{*}\right)=G^{*}\left(P_{STC,m}+k_{1}\;\ln\left(G^{*}\right)+k_{2}\;\ln{\left(G^{*}\right)}^{2}+k_{3}T^{*}+k_{4}T^{*}\;\ln\left(G^{*}\right)+k_{5}T^{*}\;\ln{\left(G^{*}\right)}^{2}+k_{6}{T^{*}}^{2}\right)$$

where the normalised in-plane irradiance $G^{*}$ and module temperature $T^{*}$ are given by equations (3), (4), respectively.

(3) $$G^{*}=\frac{G}{1000\;Wm^{-2}}$$

(4) $$T^{*}=T_{mod}-25{}^{\circ}C$$

where G is the in-plane irradiance and $T_{mod}$ is the temperature of the module. This latter is calculated using the approach suggested by Faiman [13]. Irradiance and ambient temperature values for each site were supplied by IDEAM and the National Solar Radiation Database (NSRDB) [10] through PVGIS [14]. The values for the coefficients $k_{1}$ to $k_{6}$ used in equation (2) for c-Si modules are taken from Ref. [12].

Finally, hourly solar outputs at each location were aggregated based on their installed capacities in order to obtain the solar PV distribution.

### 2.2 Electricity exports/imports dataset

The hourly electricity exports and imports distributions were built using data collected from XM [8]. The operational Colombian interconnectors are listed in Table 2. The exports/imports historical data were filtered by country and interconnector and then aggregated to obtain the total energy exchanged per hour.

**Table 2:** Electricity interconnection capacity with neighbouring countries [8].

	Import capacity [MW]	Export capacity [MW]
**Interconnection Colombia-Ecuador**		
Ecuador 230	360	500
Ecuador 138	35	35
**Interconnection Colombia-Venezuela**		
Corozo 1	55	150
Cadafe	0	36
Cuatricentenario 1	150	150
